# Frog‐biting midges and mosquitoes: Comparative insights from the Oriental and Sino‐Japanese regions

**DOI:** 10.1111/ens.70004

**Published:** 2026-02-16

**Authors:** Richa Singh, Leonardo de Campos, Ximena E. Bernal

**Affiliations:** ^1^ Department of Biological Sciences Purdue University West Lafayette Indiana USA; ^2^ Smithsonian Tropical Research Institute Ancón Panama; ^3^ Departamento de Ecologia e Zoologia, Programa de pós‐graduação em Ecologia Universidade Federal de Santa Catarina Santa Catarina Brazil

**Keywords:** amphibian‐feeding, blood‐feeding, Culicomorpha, hematophagy, host interactions, host–parasite ecological dynamics, Oriental biogeographic region

## Abstract

Frog‐biting mosquitoes (Culicidae) and midges (Corethrellidae) are old hematophagous lineages that originated over 200 million years ago and provide an ideal opportunity to broaden our understanding of the evolution of host specialization and sensory ecology. While most mosquito research has targeted medically important species, which preferentially feed on mammals and birds, a subset specializes in ectothermic hosts, particularly amphibians. Some of these species locate calling male frogs by exploiting their advertisement calls, a host‐seeking strategy that contrasts sharply with the use of chemical, thermal and olfactory cues by endotherm‐feeding species. Such interactions can influence frog signaling evolution, alter parasite transmission dynamics and shape ecological networks. Globally, understanding amphibian‐feeding Culicomorpha is critical for integrating evolutionary, ecological and conservation perspectives. Yet research is disproportionately concentrated in the Neotropics, where species diversity, host associations and behavioral adaptations have been comparatively well documented. In this review, we synthesize current knowledge on frog‐biting mosquitoes and midges in the Oriental region and compare these findings with those from Japan, as these regions share a similar amphibian lineage. A particular focus is given to India, a country hosting high anuran biodiversity hotspots, making it an ideal setting to study the ecology and evolution of frog‐biting midges and mosquitoes. By providing an overview of the status of our knowledge of these groups in the Oriental region, we identify gaps to stimulate future research. Ultimately, this review offers a foundation for researchers to develop projects focusing on fertile research venues that will advance our understanding of frog‐biting mosquitoes and midges.

## Graphical abstract

This review synthetizes current knowledge of frog‐biting mosquitoes and midges in the Oriental region and Japan, revealing strong geographic biases in research despite shared amphibian lineages. We highlight key knowledge gaps to guide future studies.

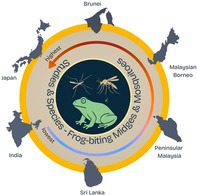

## INTRODUCTION

Hematophagy occurs in all extant families in the infraorder Culicomorpha except the solitary midges, Thaumaleidae (Lehane 2005). Feeding from amphibians, however, only occurs in three of those families: Culicidae (mosquitoes), Corethrellidae (frog‐biting midges) and Ceratopogonidae (no‐see‐ums) (Campos *et al*. 2026). While the origin and evolution of blood‐feeding behaviors in Culicomorpha are unclear (Lehane 2005), recent phylogenomic evidence suggests an amphibian‐feeding ancestor in mosquitoes (Soghigian *et al*. 2023). Within mosquitoes, some species are generalists that opportunistically feed on amphibians, but other species specialize in this clade (Campos *et al*. 2026). In contrast, the sister clade of mosquitoes, Corethrellidae, is specialized on biting frogs and toads (McKeever 1977; Bernal *et al*. 2006; Borkent 2008; Camp & Irby 2017). Some species of Culicidae and most Corethrellidae are specialized in eavesdropping on their hosts by using the mating calls of male frogs to locate them (McKeever & Hartberg 1980; Lehane 2005; Bernal *et al*. 2006; Borkent & Belton 2006; Bartlett‐Healy *et al*. 2008; Reeves *et al*. 2018; Pantoja‐Sánchez *et al*. 2023; Reinhold & Lahondère 2024; Singh *et al*. 2024). Even though specialized frog‐biting behavior has been described in species in the family Ceratopogonidae, little is known about feeding from amphibians in this group (Campos *et al*. 2026).

Considering that amphibians are both globally widespread and regionally patterned, they are an ideal system for investigating biogeographic effects on their interactions, such as those with frog‐biting flies. Contemporary delineations of amphibian geographic realms reveal sharp regional turnover and historically distinct faunas (Holt *et al*. 2013). For instance, several southern landmasses, such as the Neotropics and Australia, show sharper lineage breaks. Notably, most studies on frog‐biting mosquitoes and midges have emerged from Central and South America (Bernal *et al*. 2006; Borkent 2008; de Silva *et al*. 2015; Virgo *et al*. 2019; Leavell *et al*. 2022), regions with diverse anuran lineages. In contrast, in the Oriental region and its nearby islands, including Japan, major lineages of amphibians are often shared, even when local species differ, reflecting past land connections and migration routes across northern Eurasia (Holt *et al*. 2013). Given that the Oriental region and Sino‐Japanese region form a species‐distinct yet clade‐cohesive realm, it provides a unique opportunity to compare fly–frog interactions in regions with similar anuran lineages but different species composition.

Due to its high biodiversity, the Oriental region has the potential for hosting a wide variety of frog‐biting midges and mosquitoes. Countries within this region, such as India, Sri Lanka, Brunei and Malaysia, are home to extensive tropical rainforests and harbor a rich diversity of amphibian species, providing favorable ecological conditions for a wide array of Culicomorpha taxa feeding on these hosts. While mosquito surveillance efforts in these areas are common, they have largely concentrated on medically important genera, including *Anopheles, Aedes* and *Culex* (Tyagi 2024). Understanding the broader ecological roles of these insects in these regions, particularly their interactions with nonhuman hosts, and how such relationships could contribute to ecosystem functioning beyond the scope of human disease transmission, is important. As a first step in that direction, this review synthesizes current knowledge of frog‐biting mosquitoes and midges in the Oriental region, drawing on findings from India and neighboring countries. We also compare these findings with data from Japan, a country with similar amphibian lineages. By examining biodiversity patterns and contrasting methodological approaches, we aim to identify critical gaps and propose a framework for future research on amphibian‐feeding Culicomorpha species and their ecological interactions with these hosts across underexplored regions.

## METHODS

### Literature review

We performed a systematic literature review using the PRISMA guidelines (Moher *et al*. 2009; Page *et al*. 2021) to locate and assess research on Scopus and Web of Science. We first focused on identifying relevant studies from across the world and then narrowed down the list of studies to those from our target geographic areas (the Oriental region and Japan). Relevant studies were identified using keywords related to foraging strategies of amphibian‐attacking Diptera, including variations of associated taxonomic group names in abstracts, titles and author‐provided keywords. We only considered peer‐reviewed articles that documented interactions between Diptera and amphibians, without limiting the publication year or language. Information was gathered from the main text, tables, figures and supplementary materials, and we reached out to authors when species names were not provided. Initially, 797 documents were identified (as of June 18, 2022). An extra 261 cross‐references were examined. After eliminating duplicates and non‐peer‐reviewed articles, 724 studies advanced to the initial screening, where 337 were excluded for not addressing Diptera–amphibian interactions directly. A second screening involved full‐text evaluations using predefined criteria (Fig. S1), resulting in 235 studies. Assessing for eligibility resulted in 115 studies. From those, 15 studies were performed in countries in the Oriental region and Japan. An additional search was performed on August 31, 2025, to identify studies published since our original search, which revealed one additional study published in the last 3 years and resulted in a total of 16 studies included in the final analysis (Fig. S1). Even though ceratopogonids feed on amphibians in other parts of the world, none of the studies from the Oriental region or Japan reported species from this family. Therefore, this review focuses on flies feeding from amphibians from the other two Culicomorpha families using this strategy: Corethrellidae and Culicidae. Similarly, while mosquitoes and midges feed from urodeles (salamanders and newts) in other parts of the world, the studies identified here only report interactions with anurans (frogs and toads).

### Species richness

To evaluate species richness, we compiled the total number of Corethrellidae and Culicidae species documented across countries within the Oriental biogeographic region. Species records for each country were assembled from published literature, and unique species counts were calculated and aggregated for each region. For the Oriental region, which encompasses South and Southeast Asia, data were available from Brunei, Malaysia (subdivided into Peninsular Malaysia and Malaysian Borneo due to their distinct biogeographical characteristics), India and Sri Lanka. As a reference point, data were also obtained from Japan, from the Sino‐Japanese region (East Asia). The resulting species tallies for each fly family and region provided a basis for comparing regional diversity patterns in frog‐biting midges and mosquitoes.

### Sampling techniques

We assessed the sampling methods used to collect frog‐biting Corethrellidae and Culicidae species in countries across the Oriental and in Japan. Six techniques have been used to collect these blood‐feeding flies in the studies identified: (i) insect aspirator (a.k.a. pooters): mouth or battery‐operated aspirators to suck up and collect individual midges or mosquitoes directly from frog hosts or nearby surfaces; (ii) Center for Disease Control (CDC) light traps: standard CDC miniature light traps deployed to attract nocturnal mosquitoes; (iii) acoustic frog‐call traps: custom‐made acoustic traps consisting of a sound source broadcasting recorded frog mating calls to attract frog‐biting midges and mosquitoes; (iv) frog exposure experiments: controlled trials where live frogs are placed in enclosed arenas to attract hematophagous midges and mosquitoes; (v) sweep netting: use of insect nets to sweep through vegetation near frog‐calling sites to capture resting or flying mosquitoes and midges; and (vi) visual encounter surveys: active visual searches at night to locate flying or feeding midges and mosquitoes in the vicinity of vocalizing male frogs (e.g. hovering near the frog or sitting on the frog).

### Blood‐feeding patterns and host interactions

For each midge–frog or mosquito–frog association reported in studies from the Oriental region and Japan, we recorded a host interaction event. An interaction was defined as the presence of a particular Corethrellidae or Culicidae species on a frog individual or a frog‐baited trap. Following Grafe *et al*. (2019), each unique species–host pairing was counted as one interaction, regardless of the number of insect individuals involved. For example, multiple individuals of *Corethrella* species in a given capture event from a single frog were recorded as a single *Corethrella*–frog interaction. To characterize the blood‐feeding patterns of frog‐biting midges and mosquitoes across countries, the species interaction data were compiled into a bipartite incidence matrix representing insect species (rows) and frog species (columns), with cells indicating observed associations.

Based on country‐specific interaction matrices derived from observed associations between amphibian hosts and dipteran species, we calculated three widely used metrics: connectance, nestedness and the quantitative specialization index H2′. Connectance was measured as the proportion of realized links relative to all possible links in the bipartite network, providing a simple measure of network density and interaction breadth. Nestedness, a descriptor of interaction pattern structure, was calculated to assess the extent to which specialist species interact with subsets of the partners of more generalist species, indicating potential robustness and redundancy in the network. The quantitative specialization index H2′, which ranges from 0 (no specialization) to 1 (maximum specialization), was computed following Blüthgen *et al*. (2006) to provide a standardized estimate of network‐level specificity, accounting for interaction frequencies and sampling completeness. All network analyses were performed using the bipartite package in R (version 2.17), following Dormann *et al*. (2008) and Blüthgen *et al*. (2006).

## RESULTS

### Literature review

**Figure 1:**
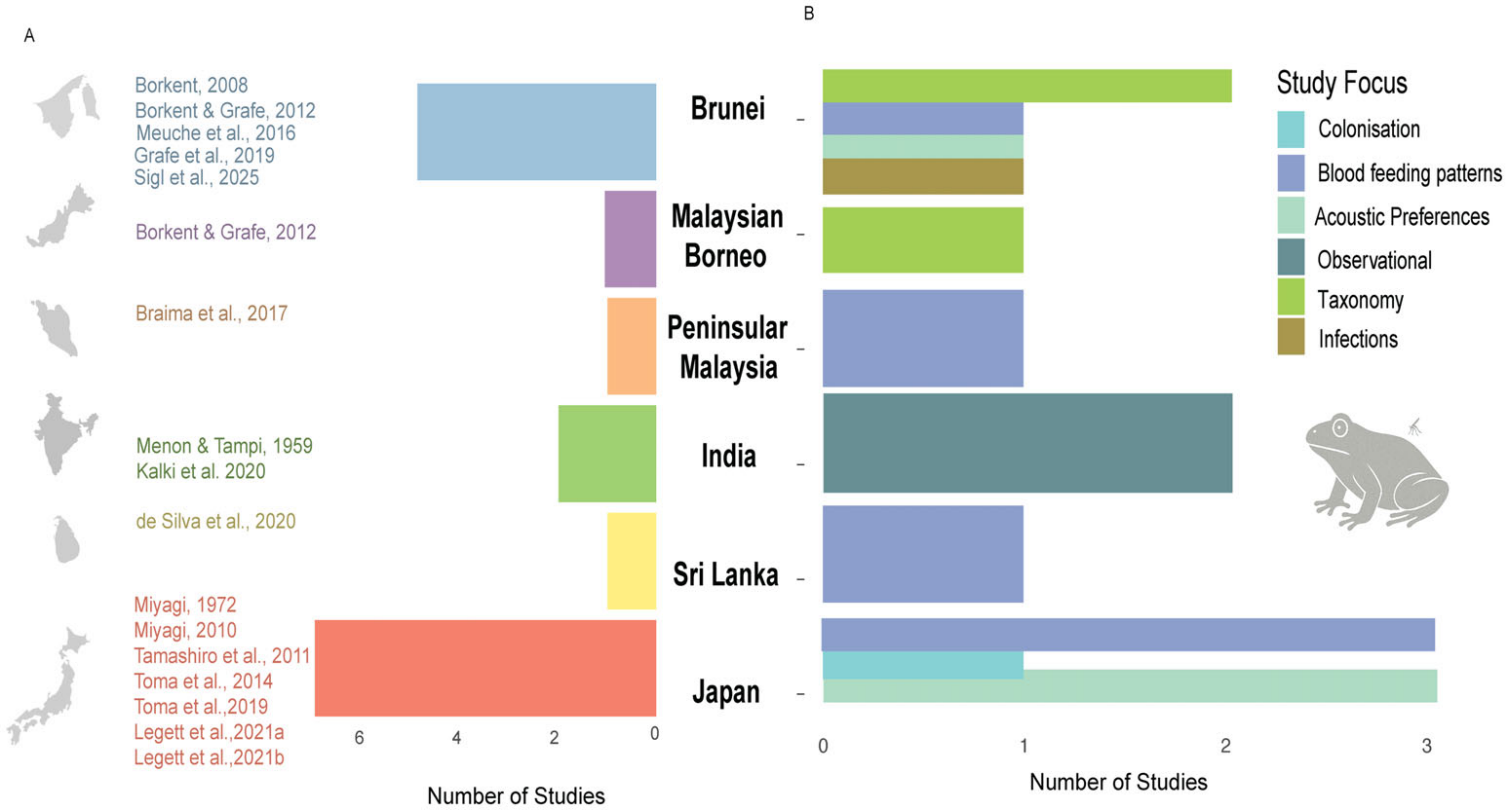
Studies from different countries in the Oriental region and Japan (Sino‐Japanese region) investigating frog‐biting mosquitoes (Culicidae) and midges (Corethrellidae). (A) Number of studies conducted in each country. (B) Focus of the studies reported across different countries. Colonization, exposing frogs to establish a colony in the laboratory; Blood‐feeding patterns, acoustic trap experiments designed and blood meal identification done for confirming blood feeding; Acoustic Preferences, acoustic trap experiments to examine the preference for frog‐call features; Observational, visual search at night for locating flies hovering near or sitting on the frog; Taxonomy, species identification and description.

The distribution of published studies on frog‐biting Corethrellidae and Culicidae within the Oriental region is uneven, with the highest number of records from Borneo (Brunei *n* = 5, Malaysian Borneo *n* = 1), and relatively few studies from the other areas (Peninsular Malaysia *n* = 1, India *n* = 2 and Sri Lanka *n* = 1; Fig. 1A). In Japan, from the Sino‐Japanese region, seven studies have been conducted. The earliest work in Japan dates to 1972, with sustained research activity from 2008 to 2021. In Brunei, studies were conducted between 2008 and 2025, while the single Sri Lankan study was published in 2020. In India, the first report appeared in 1959, followed by an 81‐year gap before the next record in 2020 (Fig. 1A).

The research focus of these studies also varies geographically (Fig. 1B). Research from Brunei has addressed a broad range of topics, including taxonomy, blood‐feeding patterns, acoustic responses to frog calls and the specificity of *Trypanosoma*, a blood parasite transmitted by corethrellid midges. A study from Malaysian Borneo identified and described four midge species (Borkent & Grafe 2012), while studies from Peninsular Malaysia and Sri Lanka examined blood‐feeding patterns only in frog‐biting mosquitoes (Braima *et al*. 2017; de Silva *et al*. 2020). Similarly, in Japan, diverse research topics have been investigated, covering blood‐feeding patterns, acoustic preferences and colonization of frog‐biting species. In contrast, in India, studies so far have been limited to visual encounter records (Fig. 1B).

### Species richness

**Figure 2:**
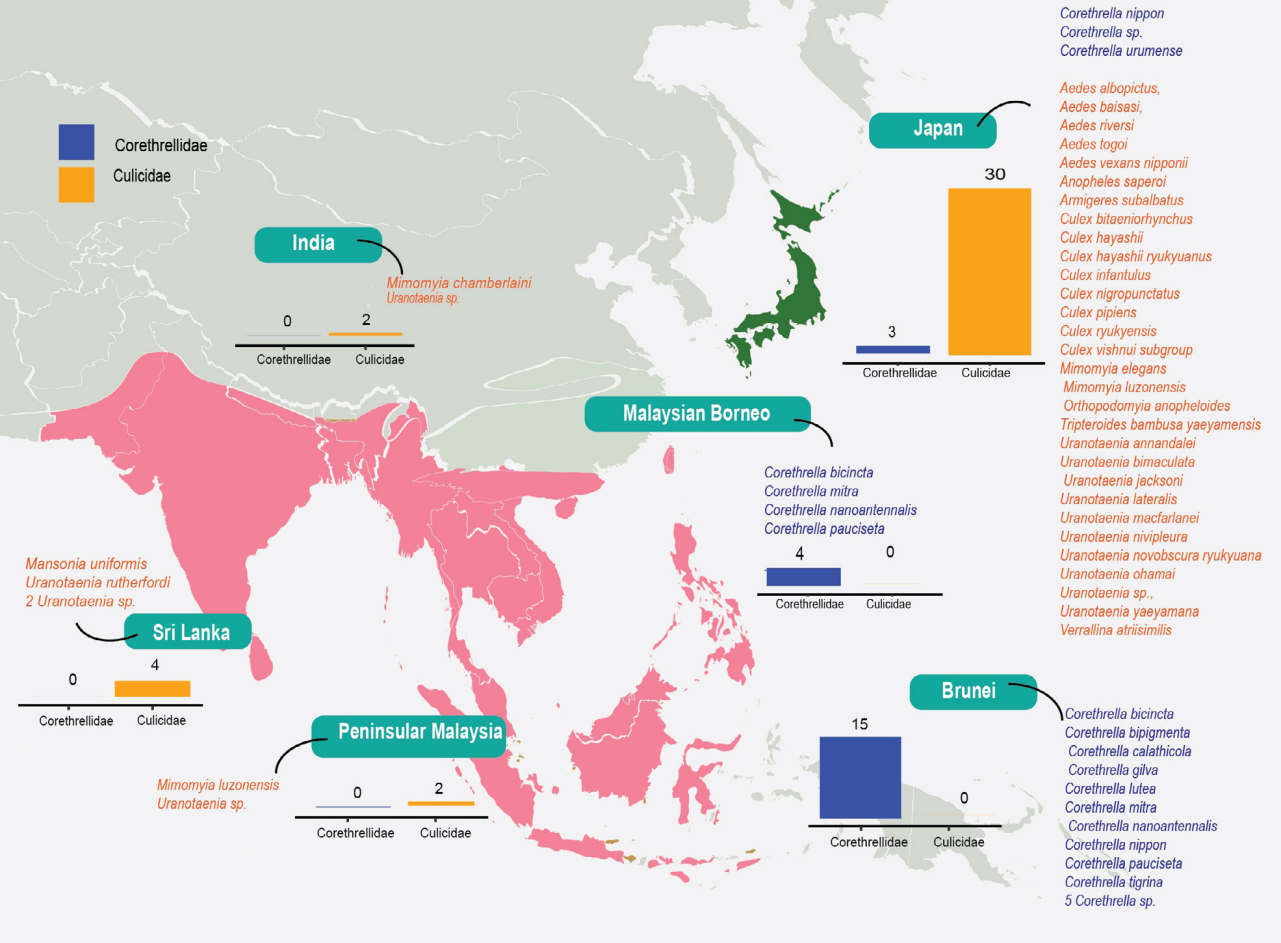
Frog‐biting mosquitoes (Culicidae) and midges (Corethrellidae) reported across the Oriental region (pink) and Japan (green), in the Sino‐Japanese region. The bars represent the number of species reported to feed from anurans from these two families (Culicidae in orange; Corethrellidae in blue). The species names listed alongside indicate the reported species across studies for each area.

In the Oriental region, records of frog‐biting Corethrellidae are limited to Brunei (15 species) and Malaysian Borneo (4 species), while frog‐biting Culicidae are documented in Peninsular Malaysia (2 species), India (2 species) and Sri Lanka (4 species) (Fig. 2). In contrast, in Japan, species from both families, with 30 species of frog‐biting Culicidae and 3 species of Corethrellidae, have been reported (Fig. 2).

### Sampling techniques

**Figure 3:**
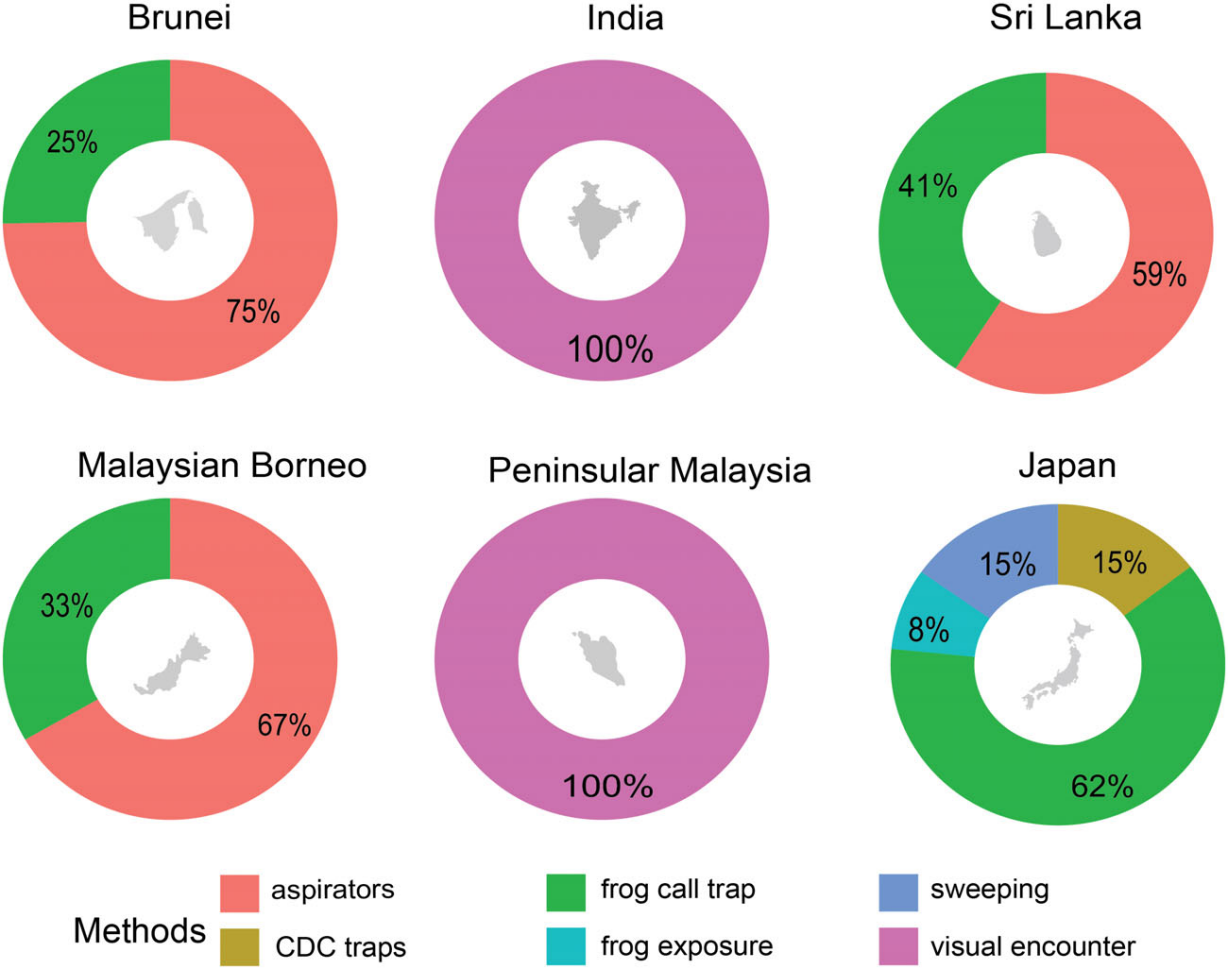
The diverse techniques used to study the associations between frogs and midges and mosquitoes across countries in the Oriental region and Japan. Donut plots show the percentage of different sampling methods used across countries (aspirator, sweeping, frog exposure visual encounter, frog‐call trap, CDC, etc.). The methods are described in the text.

In the Oriental region, aspirator‐based collection was the predominant method for sampling midges and mosquitoes (Fig. 3). It accounted for the majority of records in Brunei (75%), Malaysian Borneo (67%) and Sri Lanka (59%). The only other method employed in these countries was the use of frog‐call traps, which have been deployed in Brunei (25%), Malaysian Borneo (33%) and Sri Lanka (41%) (Fig. 3). In contrast, visual encounter surveys were the sole technique reported from India and Peninsular Malaysia (Fig. 3). In Japan, researchers employed a broader range of sampling techniques, reflecting a more diversified strategy: frog‐call traps were the most frequently used (62%), followed by CDC light traps (15%), sweeping (15%) and direct exposure of frogs under controlled conditions (8%).

### Blood feeding patterns

**Figure 4:**
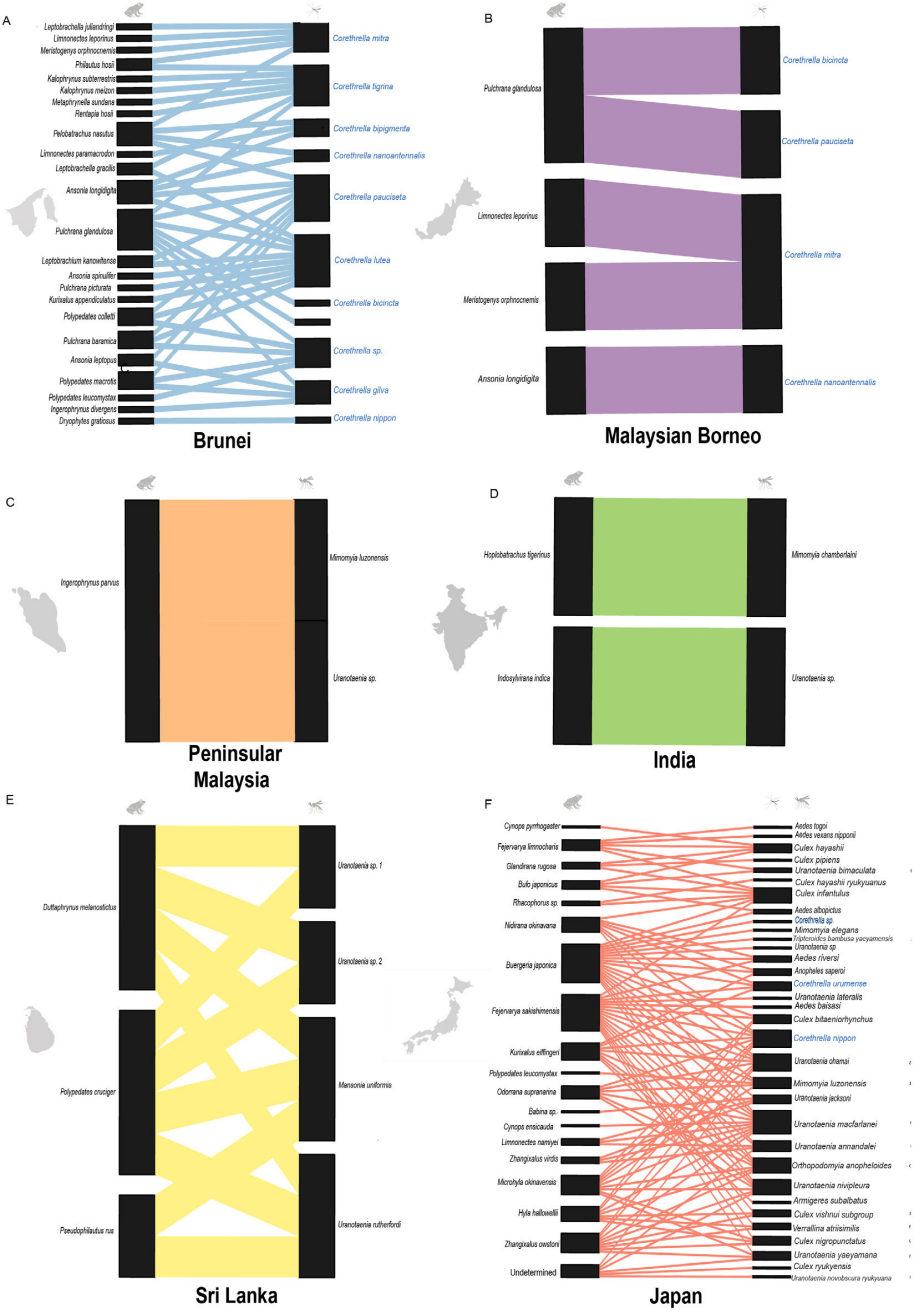
Blood‐feeding patterns of frog‐biting mosquitoes (Culicidae) and midges (Corethrellidae) across countries in the Oriental region and Japan. Each alluvial diagram shows the frog species in the left column and the Diptera species (Culicidae in black font; Corethrellidae in blue font) in the right column. The thickness of the lines connecting flies and frogs indicates the strength of the association between those groups. Different line colors are used for different countries where these interactions occur.

We recorded a total of 198 interactions between frog‐biting Corethrellidae and Culicidae and anurans across all surveyed countries. Of these, 129 interactions originated from Japan, while the remaining 69 interactions were recorded from Oriental region countries (Fig. 4). As mentioned before, no species of Ceratopogonidae feeding on amphibians has been reported for these countries, and only interactions with anurans have been recorded to date.

Within the Oriental region, Brunei exhibited the highest interaction richness, with 49 recorded interactions involving 24 frog species and 11 midge species, making it the top‐ranked country in terms of species diversity and interaction frequency (Fig. 4A). Malaysian Borneo yielded five interactions between four frog species and four midge species (Fig. 4B), whereas Peninsular Malaysia recorded three interactions involving one frog species and two mosquito species (Fig. 4C). In Sri Lanka, we identified 10 interactions involving three frog species and four mosquito species (Fig. 4E). India had the lowest number of records within the countries examined in the Oriental region, with only two interactions involving two frog species and two mosquito species (Fig. 4D). In contrast, Japan accounted for the majority of records, with 129 interactions (65%) encompassing 19 frog species, 30 mosquito species and three midge species (Fig. 4F). Analysis of the interaction networks revealed high variation in connectance (proportion of realized to potential links) with countries with high connectance in Peninsular Malaysia (1.00) and Sri Lanka (0.83), intermediate connectance in India (0.50) and low connectance in Brunei (0.17), Malaysian Borneo (0.31) and Japan (0.18). Nestedness (the degree to which specialist interactions are subsets of generalist ones) also varied, with high nestedness in Malaysian Borneo (40.0) and India (37.7), intermediate nestedness in Brunei (19.1) and Japan (15.0), low nestedness in Sri Lanka (0.032) and nil in Peninsular Malaysia (0).

## DISCUSSION

This review synthesizes current knowledge on the diversity and host interactions of frog‐biting mosquitoes (Culicidae) and frog‐biting midges (Corethrellidae) across the Oriental biogeographic region and Japan, providing the first perspective on the status of research on these families in these areas. Collectively, the available literature reveals a clear imbalance in research effort, with substantial geographic disparities in species records despite the broad distribution of shared amphibian lineages across these regions. These disparities shape our current understanding of species diversity, host use and ecological interactions and highlight the influence of both sampling bias and methodological inconsistencies in present regional knowledge.

### Regional gaps in frog‐biting mosquito and midge research

Research in the Oriental region is disproportionately concentrated in Brunei, where systematic, long‐term surveys have documented the highest known Corethrellidae richness in this area. The use of quantitative community‐level approaches, particularly bipartite host–parasite network analyses, has facilitated comparisons of specialization patterns across mixed‐dipterocarp and peat swamp forest habitats, highlighting how habitat structure shapes fly–frog interactions (Borkent 2008; Borkent & Grafe 2012; Meuche *et al*. 2016; Grafe *et al*. 2019). Similar habitat‐driven variation in host attraction has been described in the relationships between corethrellids and frogs in Malaysian Borneo (Borkent & Grafe 2012). Playback experiments used to assess host use further highlight the role of acoustic cues in mediating host attraction, which is linked to trypanosome infection risk (Meuche *et al*. 2016). This blood parasite, associated with corethrellids in both Borneo and the Americas, is diverse and anuran‐specific, occurring in an infective, host‐competent stage (Sigl *et al*. 2025).

The concentration of detailed research in Brunei contrasts sharply with the limited and sporadic sampling elsewhere in the Oriental region. Peninsular Malaysia, Sri Lanka and especially India rely heavily on observational records or limited trapping sessions. Such restricted sampling can (i) artificially exaggerate species‐level specialization, (ii) obscure seasonal or habitat‐driven variation and (iii) limit inference on ecological or evolutionary processes. A study performed in Sri Lanka provided deep insights into temporal and spatial species‐specific synchronization between mosquito foraging and frog calling activity, while also revealing new records of *Uranotaenia* species (de Silva *et al*. 2020), but such comprehensive sampling remains geographically narrow. In Peninsular Malaysia, the research has been observation‐based near an oil palm plantation settlement, resulting in records of *Mimomyia* (*Etorleptiomyia*) *luzonensis* feeding on a toad, *Ingerophrynus parvus* (Braima *et al*. 2017). This description identifies host use in human‐modified landscapes, but lacks a broader ecological context, emphasizing the need to examine frog‐biting fly communities in natural and altered environments to assess how anthropogenic changes affect these clades and their host associations.

India remains exceptionally under sampled. The few records available are largely the result of opportunistic sightings spread over nearly a century. The record of *Mimomyia chamberlaini* feeding on the Indian bullfrog (*Rana tigrina*) dating to the early 20th century (Menon & Tampi 1959) was followed by another isolated field sighting of *Uranotaenia* sp. on an Indian Golden‐backed Frog (*Indosylvirana indica*) (Kalki *et al*. 2020). The long interval without studies on frog‐biting mosquitoes or midges highlights the limited interest and investment in these groups in this country, which has hindered the discovery of species diversity and host–parasite relationships in India.

In contrast, in Japan, studies have implemented diverse methodological approaches integrating behavioral experimentation, acoustic manipulation, molecular identification and diverse trapping strategies (Miyagi 1972; Miyagi *et al*. 2010; Tamashiro *et al*. 2011; Toma *et al*. 2014, 2019; Legett *et al*. 2021a,b). Studies from the Ryukyu Archipelago, for instance, provide detailed information on the natural history of *Uranotaenia macfarlanei* (Miyagi *et al*. 2010) and experimental work investigating the responses of *Uranotaenia* mosquitoes and *Corethrella* midges to natural and synthetic calls of common frog species in the Ryukyu Archipelago (*Fejervarya sakishimensis, Microhyla okinavensis, Rana okinavana, Rhacophorus owstoni, Odorrana supranarina, Kurixalus eiffingeri* and *Buergeria japonica*). These studies have shown how mosquito and midge host attraction is shaped by acoustic complexity, amplitude and call timing between neighboring frogs (Toma *et al*. 2019; Legett *et al*. 2021b). These acoustic responses also suggest fly‐mediated selection may have driven call synchronization among neighboring males in the chorus, making flies active participants in the evolutionary trajectory of frog communication (Legett *et al*. 2021a).

Bloodmeal analysis using PCR has further revealed strong amphibian specialization by several Japanese *Uranotaenia* species (99.1%), emphasizing tight ecological coupling between certain mosquito lineages and anuran hosts in this region (Toma *et al*. 2014). Surveys using frog‐call traps, black light traps and sweeping nets in five Japanese islands at the Ryukyu Archipelago, followed by bloodmeal analysis of the specimens collected, yielded a comprehensive list of 12 species from four mosquito genera: *Aedes*, *Culex*, *Mimomyia* and *Uranotaenia* (Tamashiro *et al*. 2011). These findings reinforce the ecological significance of frogs as blood sources in multi‐host systems, including species that feed on endotherms. The combination of behavioral experimentation, molecular confirmation of host use and diverse trapping strategies has enabled Japanese researchers to document species richness, host choice and behavioral interactions in frog‐biting mosquitoes and midges across five islands in the Ryukyu Archipelago in southern Japan: Amami‐Oshima, Tokunoshima, Iheya, Okinawa and Iriomote (Miyagi 1972; Miyagi *et al*. 2010; Tamashiro *et al*. 2011; Toma *et al*. 2014, 2019; Legett *et al*. 2021a,b). Higher species richness and more diverse host interactions, however, are expected as other Japanese regions are investigated.

### Cross‐regional patterns in fly–frog interaction networks

The comparison of network metrics across countries collectively illustrates variation in our understanding of frog–fly associations, revealing limitations of current data. In countries where sampling is extensive (e.g. Japan and Brunei), networks appear diffuse, with many rare interactions. Japan and Brunei have the highest species richness and show low connectance, indicative of broad but diffuse interaction webs in which many potential links are realized only infrequently. Such network structures are typical of well‐sampled, species‐rich systems where interactions are distributed across a diverse host and parasite community (Grafe *et al*. 2019; Virgo *et al*. 2022). Where sampling is limited (e.g. Sri Lanka, Peninsular Malaysia, India), the networks are narrower and more cohesive, likely inflated by data scarcity. Sri Lanka and Peninsular Malaysia exhibit very high connectance coupled with almost zero nestedness, reflecting strong species‐specific host associations with minimal overlap among mosquito or midge species in their choice of frog hosts. However, the connectance scores of Sri Lanka and Peninsular Malaysia are based on only 10 and three recorded interactions, respectively, which likely overestimate network cohesion. Similarly, Malaysian Borneo and India display intermediate patterns likely influenced by incomplete sampling. Thus, patterns of connectance and nestedness across regions must be interpreted with caution, as they can reflect sampling effort rather than underlying ecological structure. Further research that involves long‐term, systematic sampling efforts, similar to those from studies in Japan and Brunei, is necessary to capture the complex and diffuse structure of interactions between flies and frogs in these areas.

### Priorities for future research

India represents one of the largest knowledge gaps in the region. Despite being a global amphibian biodiversity hotspot (Dinesh 2024), Indian records of frog‐biting mosquitoes and midges are limited to isolated sightings across nearly a century. The absence of systematic surveys hampers any meaningful ecological or evolutionary inference. Given that research in India has long focused on medically important mosquitoes (Tyagi 2024), targeted studies of mosquitoes and midges that exploit anurans are needed to fill the wide current gap in knowledge about the ecological relationships between these flies and their hosts in this country.

Addressing this gap will benefit from coordinated, long‐term sampling with multipronged approaches integrating standardized acoustic trapping (Bernal *et al*. 2006; Toma *et al*. 2019; de Silva *et al*. 2020; Legett *et al*. 2021b), and use of molecular tools such as DNA barcoding and *COI* sequencing to detect cryptic fly diversity (Virgo *et al*. 2022). Taxonomic and descriptive studies will also provide the foundation to develop hypothesis‐driven research, further broadening our understanding of these fly families. Moreover, studies focusing on how anthropogenic changes may disrupt the interactions between flies and anurans would be valuable. India, like other growing economies, has experienced rapid urbanization in the recent past (Jiang & O'Neill 2017). Studies investigating how such habitat degradation influences fly and frog species distribution, abundance and host–parasite dynamics, building on evidence from other regions showing corethrellid midges’ sensitivity to artificial light at night and noise pollution (McMahon *et al*. 2017), would contribute to fly–frog interactions in a rapidly changing environment.

India has diverse amphibian lineages that include 450 species, with high endemism in the Western Ghats, Eastern Himalayas and other diversity hotspots (Dinesh 2024). These areas include endemic frog species such as the purple frog, *Nasikabatrachus sahyadrensis* (Thomas *et al*. 2014), bush frogs of the genus *Raorchestes* (Vijayakumar *et al*. 2014) and tree hole breeding frogs from the genus *Frankixalus* (Biju *et al*. 2016). The high incidence of endemic species presents exceptional opportunities to investigate fly diversification, host specialization and biogeographic history. Comparative studies with Sri Lanka, given their shared geological history, could also provide valuable insights about the evolutionary history and potential host divergence of fly lineages separated for more than 130 million years. We hope this review will stimulate cross‐country collaborations that will take advantage of this unique, natural setting to contrast fly–frog associations across the Palk Strait.

### Broader Global Context and Implications

Overall, the low and uneven distribution of studies investigating frog‐biting Corethrellidae and Culicidae in India and across the Oriental biogeographic region reflects a yet to be explored gap that is likely to provide insightful lessons for our understanding of these groups. In addition, this gap reflects how our global understanding of frog‐biting Corethrellidae and Culicidae is disproportionately shaped by a few world regions, potentially obscuring broader patterns in host–parasite ecological dynamics and evolutionary patterns. For instance, our current understanding of frog‐biting midges comes mainly from studies in the Neotropics, where most species from this family have been described (Borkent 2008; Amaral *et al*. 2025; Campos *et al*. 2026). Furthermore, in that region, hypothesis‐driven behavioral studies have examined host use (Virgo *et al*. 2022), activity patterns relative to frog choruses (Virgo *et al*. 2019, 2022) and dissected acoustic preferences to frog calls that may shape signaling evolution (Bernal *et al*. 2006; de Silva *et al*. 2015; Legett *et al*. 2020; Leavell *et al*. 2022). Whether similar patterns occur in the Oriental region remains largely unknown due to uneven sampling and insufficient implementation of experimental and molecular approaches.

By highlighting regional differences in sampling effort, methodological approaches and key knowledge gaps, this review aims to promote integrative research that situates frog‐biting Corethrellidae and Culicidae within broader ecological and evolutionary frameworks in the Oriental region. Cross‐country collaborations, standardized sampling protocols and expanded molecular and behavioral studies will be essential for uncovering the full diversity of these fly families and understanding the evolution of their unique feeding strategies.

## CONFLICT OF INTEREST STATEMENT

The authors declare no competing or financial interests.

## Supporting information


**Figure S1.** PRISMA flow diagram for systematic review.

## Data Availability

The data that supports the findings of this study are available in the supplementary material of this article.
