# Supplementary material for: Frog‐biting midges and mosquitoes: Comparative insights from the Oriental and Sino‐Japanese regions
Source: Entomol Sci. 2026 Feb 16;29(1):e70004. doi: 10.1111/ens.70004 (PMC12908430; doi:10.1111/ens.70004)

Frog-Biting Midges and Mosquitoes in India: Comparative Insights from the Oriental Region

S1. PRISMA flow diagram for systematic review.


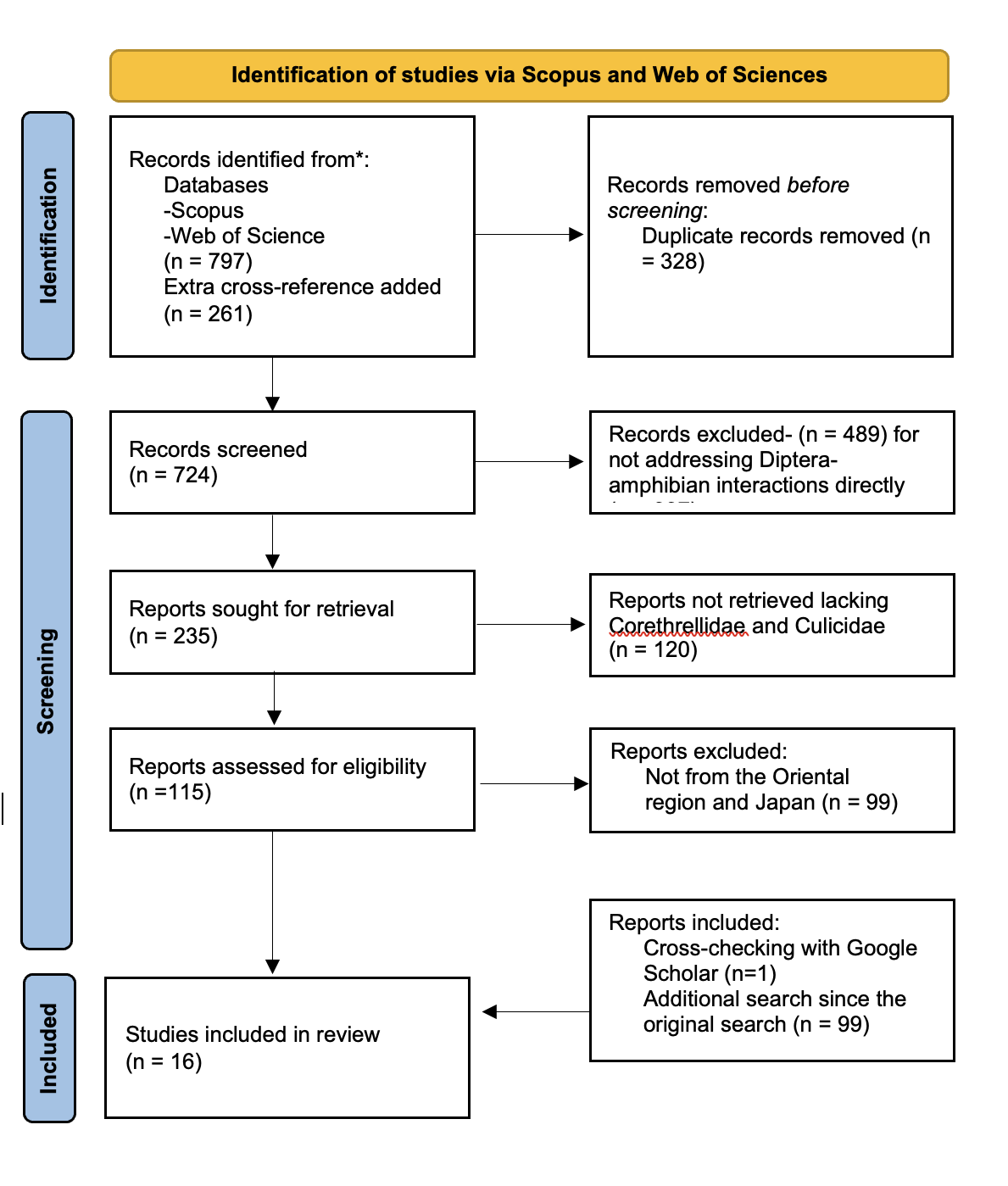

Supplement: Supplementary file 1 — Figure S1. PRISMA flow diagram for systematic review. [file ENS-29-e70004-s001.docx]
